# Physician Satisfaction With Lactation Resources Following an Intervention to Improve Lactation Accommodations

**DOI:** 10.1001/jamanetworkopen.2023.27757

**Published:** 2023-08-08

**Authors:** Michelle Mourad, Priya Prasad, Caroline Wick, Diane Sliwka

**Affiliations:** 1 School of Medicine, University of California San Francisco; 2 Lactation Accommodation Program, Campus Life Services, University of California San Francisco

## Abstract

**Importance:**

Physicians who attempt to continue breastfeeding after returning from childbearing leave identify numerous obstacles at work, which may affect job satisfaction, retention, and the diversity of the physician workforce.

**Objective:**

To study the association between improved lactation accommodation support and physician satisfaction.

**Design, Setting, and Participants:**

This cohort study compared the physician experience before and after a July 2020 intervention to improve physician lactation accommodation support at a large, urban, academic health system. The satisfaction of physicians returning from childbearing leave between July 1, 2018, and June 30, 2020 (preintervention), was compared with that of physicians returning from leave between July 1, 2020, and November 30, 2021 (postintervention). Initial data analysis was performed on February 22, 2022, with additional tests for interaction performed on May 18, 2023.

**Intervention:**

The intervention included creating functional lactation spaces, redesigning communication regarding lactation resources, establishing physician-specific lactation policies, and developing a program to reimburse faculty for time spent expressing breastmilk in the ambulatory setting.

**Main Outcomes and Measures:**

The main outcomes were (1) space improvements, use, and costs of the lactation accommodation program and (2) an ad hoc survey of physicians’ reported experience with lactation accommodation support before and after the intervention. Survey data were collected using a 5-point Likert scale to assess physician perceptions of institutional support. Responses collected during the preintervention period were compared with those collected during the postintervention period using unpaired *t* tests.

**Results:**

In this study, 70 clinical faculty (mean [SD] age, 34.4 [2.9] years) took childbearing leave in the preintervention period compared with 52 (mean [SD] age, 34.8 [2.7] years) in the postintervention period. Fifty-eight physicians (83%) completed the preintervention survey and 48 completed the postintervention survey. When comparing the pre- and postintervention periods, faculty reported improvements in finding time in their clinical schedule to devote to pumping (mean [SD] response, 2.5 [1.3] vs 3.6 [1.5]; *P* < .001), initiatives to address the impact of lactation time on productivity (mean [SD] response, 2.0 [1.0] vs 3.0 [1.5]; *P* = .001), and a culture supportive of lactation (mean [SD] response, 2.8 [1.4] vs 3.4 [1.3]; *P* = .047). Forty childbearing faculty took advantage of lactation time reimbursement and were reimbursed a total of $242 744.37. Faculty whose return to work overlapped with the entire year of the study received financial support for lactation for a mean (SD) of 8.9 (0.2) months, with an average reimbursement of $9125.78.

**Conclusions and Relevance:**

The findings of this cohort study suggest that a multifaceted intervention to combat common challenges in lactation support in academic medical centers yielded improvements in faculty perceptions of institutional support for pumping breastmilk, addressing the impact of lactation time on productivity, and providing a culture supportive of lactation. These findings support the adoption of interventions to improve physician lactation accommodations.

## Key Points

**Question:**

Can an intervention to improve lactation accommodations at a large US academic health system, including financial reimbursement for time spent expressing breastmilk, address known barriers to physician breastfeeding?

**Findings:**

In this cohort study of 122 physicians returning from childbearing leave, participants reported improvements in finding time in their clinical schedule to devote to pumping breastmilk, initiatives to address the impact of lactation time on productivity, and a culture supportive of lactation. In the first program year, 40 physicians obtained financial reimbursement for time spent expressing breastmilk, costing the health system $242 744.

**Meaning:**

These results suggest that improving lactation accommodations in academic health systems could remove known barriers to physician lactation.

## Teaser

This cohort study examines the association between improved lactation accommodation support and physician satisfaction at a large, urban academic health system.

## Introduction

Representation of female physicians in academic medicine is increasing. A previous study reported that 41% of full-time medical school faculty and approximately 46% of medical school graduates are women.^1^ Despite this growing representation, equitable policies around lactation accommodations remain a challenge and may contribute to premature breastfeeding cessation. One study of the challenges of continuing to breastfeed in academic medicine reported that while 97% of childbearing physicians reported breastfeeding initiation and 64% reported an intention to breastfeed at least 12 months, only 41% were still breastfeeding at 1 year.^2^ The gap is more pronounced for medical trainees, with one study reporting that 29.5% were breastfeeding at 6 months.^3^

Lactating physicians who attempt to maintain breastfeeding after parental leave identify numerous obstacles at work: most notably, lack of both sufficient time and an adequate place for milk expression.^4,5^ Their main reasons for breastfeeding cessation, especially between 1 and 12 months post partum, are work related.^4,5,6,7^ In a cross-sectional study of physician mothers in the US, inadequate time for pumping was the most commonly reported negative experience upon return to work from childbearing leave.^4^

Breastfeeding support is important in the retention of physician parents and in the gender equity and diversity of academic medicine. Financial losses and inflexible work schedules have been shown to affect career satisfaction among women.^8,9,10^ In much of academic medicine, physicians who choose to breastfeed may have to work harder or work more hours to make up for time lost to pumping or associated bottling and washing of pump parts.^4,5,6^

Although the Patient Protection and Affordable Care Act amended the Fair Labor Standards Act to require employers to provide “reasonable break time for an employee to express breast milk for her nursing child for 1 year after the child’s birth each time such employee has need to express the milk,”^11^ these protections do not require that employers compensate employees for their time in jobs where productivity can affect pay and bonuses. At the University of California San Francisco (UCSF) School of Medicine, physician productivity is largely measured in work relative value units (wRVUs),^12^ and lactating physicians may appear to be less productive than their nonlactating counterparts and are at risk for lost wages or bonus payments. Therefore, we aimed to improve the experience and culture around lactation throughout our institution and adopted a program to financially reimburse ambulatory physicians for their time spent expressing breastmilk. We describe the implementation of these efforts, evaluate costs, and use a pre- to postimplementation analysis of the experiences of lactating physicians to assess the association between improved lactation accommodation support and physician satisfaction.

## Methods

### Study Setting

The UCSF School of Medicine employs 3000 physician faculty working at UCSF Health, of which 1260 (42%) identify as women. The UCSF Health system provides broad benefits for all faculty, including 12 weeks of paid childbearing or childrearing leave. The UCSF institutional review board reviewed this cohort study and deemed it exempt from review and the need for informed consent given the primary aim of improving the physician experience. The study followed the Strengthening the Reporting of Observational Studies in Epidemiology (STROBE) reporting guideline.

Annually, UCSF Health surveys all faculty using the Net Promotor Score (NPS), measuring the likelihood of a faculty member recommending UCSF as a place to do clinical work.^13^ In 2019, a 20-point gap in the NPS was noted in the responses between faculty identifying as male vs female (19 vs −1). In faculty focus groups with women, return from childbearing leave was identified as one factor affecting physician satisfaction. We undertook a needs assessment and subsequent interventions to support lactation as one way to improve the experience of faculty returning from childbearing leave (Table 1).

**Table 1.** Lactation Accommodation Improvement Aims, Partnerships, and Key Contributions

Aim and partner	Key contribution
**Lactation spaces: needs assessment**	
Medical director and frontline faculty	Challenges specific to each clinical area (eg, anesthesiologists finding pumping breaks between short cases with quick turnover)
Lactation specialist	Survey of current spaces, room capacity and availability, and compliance with state and federal regulations (eg, old clinical buildings where lactation spaces do not meet regulatory requirements)
**Lactation credits: needs assessment**	
Human resources	Number and demographics of faculty over the past several years who have taken childbearing leave; obtaining approval from faculty to provide faculty names and leave dates to estimate programmatic costs
Faculty practice revenue management	Ambulatory wRVU productivity preceding childbearing leave for faculty working in the ambulatory setting
**Lactation space improvements**	
Medical director and frontline faculty	Identification of potential new lactation spaces: consultation rooms, breakrooms, clinic rooms, and offices
Lactation specialist	Knowledge of regulations regarding converting existing space to a lactation room
Health system	Commitment to funding for furniture, equipment, and security for all new lactation spaces
Facilities	Furnishing, equipment, room locks, and badge readers
Information technology	Lactation room scheduling system, phone connection, and network access for all computers
Hospitality	Scheduled twice-daily cleaning for all lactation rooms
**Lactation credit financial reimbursement**	
Faculty experience officer	Institutional experience of faculty returning from childbearing leave; opportunity cost of funding lactation wRVU reimbursement on faculty well-being compared with other planned interventions designed to improve the faculty experience
Diversity, equity, and inclusion officer	Equity for faculty who experience challenges meeting productivity targets or earning bonuses due to lactation
Faculty practice and health system	Commitment of funds to support faculty salaries for time spent expressing breastmilk
Faculty practice revenue management	Incorporation of wRVUs and reimbursement into existing faculty funding model
**Department-specific lactation accommodations**	
Clinical department chair	Commitment to department-specific improvements: local spaces, financial support for equipment, and accommodations in schedules
Chief administrative officer	Creation and communication of department-specific policies; advocacy for creation of local departmental spaces for lactation
Medical director	Incorporation of lactation accommodations into faculty clinical schedules

Abbreviation: wRVU, work relative value unit.

### Needs Assessment

We engaged medical directors and focus groups of frontline faculty across ambulatory, perioperative, and inpatient departments to discuss challenges with lactation faced by faculty. Participants in each group were asked the standard open-ended questions about what was working well with lactation accommodations, what needed to be improved, and how UCSF Health and their department or division could help support their needs. Meeting notes were analyzed and 4 key themes were identified: (1) providing information about lactation accommodations (eg, UCSF policies, pumping locations, and available equipment); (2) cleanliness, availability, functionality, and proximity of lactation spaces; (3) protecting time for lactation during clinical work and accounting for lactation in measures of productivity; and (4) a work culture that is supportive of lactation. Similar themes have been identified in previous studies examining barriers to lactation in academic medicine.^4,5,6,11^

### Institutional Commitment

We obtained a memorandum of understanding that if additional lactation spaces could be identified, UCSF Health would resource those spaces with pumps, computers, desks, and phones to meet the demand for functionality. We also received support from the committee responsible for clinician reimbursement in that, for ambulatory physicians, time spent expressing breastmilk would be reimbursed by wRVUs, conditional on an estimation of costs (eAppendix 1 in Supplement 1).

### Initiatives

#### Lactation Space, Furnishings, and Cleanliness

We partnered with our Campus Life Services lactation specialist (C.W.) to identify buildings with insufficient lactation rooms and administrators at those sites to identify potential lactation spaces. Our lactation specialist toured any identified rooms with facilities to evaluate room suitability and required enhancements (eg, installation of privacy features or purchase of equipment).

#### Ambulatory wRVU Support for Lactation Time

Although a decrease in wRVUs does not result in a change in base salary for most faculty at UCSF, the decrease in measured productivity can affect access to bonuses, scribe support, and staffing resources for their clinic. To rectify this gap, starting in November 2020, UCSF Health began supporting lactation wRVUs for ambulatory physicians. Faculty returning from childbearing leave are given the option of adding 1 lactation hold appointment to their clinic schedule per half-day session until their child is aged 1 year. Each lactation hold is reimbursed as a set amount of wRVUs, equivalent to *Current Procedural Terminology* (*CPT*) code 99214 (currently 1.92 wRVUs). Commensurate with the number of lactation holds, the faculty practice provides the wRVU count and associated funds to the clinician’s home department monthly, similar to how *CPT*-based wRVUs and resulting payments are made to faculty for clinical visits. In total, these wRVUs and funds contribute to productivity metrics and support the physician’s salary, respectively. Allied health professionals and physicians whose compensation is based on number of clinic sessions rather than wRVUs, as well as trainees, are encouraged to use lactation holds; however, no transfer of funds occurs. The program was publicized to all ambulatory physicians who took childbearing leave in the preceding 12 months and via medical directors and clinic administrators (eAppendix 2 in Supplement 1).

### Survey Instrument and Process

We developed a survey informed by the challenges to lactation reported by our focus groups and based on prior surveys in the literature.^4,5,6,11,12^ The goal of the preintervention survey was to obtain baseline satisfaction with lactation resources, whereas an identical postimplementation survey assessed physician satisfaction with our lactation improvement initiatives. The survey asked about the degree of support provided by the UCSF Lactation Accommodation Program on communication around lactation policies, lactation space availability, cleanliness, proximity, accounting for changes in clinical productivity due to pumping, and UCSF’s culture around supporting lactation, in addition to limited demographic questions (ie, age, department, clinical setting, location, and length of faculty appointment). All survey questions underwent review for clarity and comprehension by pretesting among a group of physician stakeholders (eAppendix 3 in Supplement 1).

Both the preintervention and postintervention surveys aimed to capture all clinical faculty returning from childbearing leave. Prior to implementation, the survey was sent to all 70 clinical faculty who took childbearing leave at UCSF Health in the 2 years before implementation (between July 1, 2018, and June 30, 2020). Postimplementation (between July 1, 2020, and November 30, 2021), we sent our survey to those known to have used the lactation wRVU credit program between November 2020 and November 2021, and we asked all clinical departmental administrators to send the survey to clinical faculty in their department who had returned from childbearing leave in the 2021 academic year. This helped ensure that nonambulatory faculty who may have not been eligible for the lactation wRVU credit program participated in the survey. The surveys were sent with an anonymous link by email, with 3 reminders.

### Statistical Analysis

Survey data were collected using a 5-point Likert scale to assess physician perceptions of institutional support, with 5 indicating great support. The data were summarized using means (SDs), and responses collected during the preintervention period were compared with those collected during the postintervention period using unpaired *t* tests for the cohort as a whole and for subgroups defined by length of faculty appointment (≥5 years vs <5 years). Given the dissemination method for the postimplementation survey, we were not able to calculate a response rate; however, we did obtain limited demographic information from human resources on the number of physicians returning from childbearing leave in the 2021 academic year. Although it is possible that the same individual answered the survey in both the pre- and postintervention periods (eg, took childbearing leave in both periods), we were unable to pair the responses. All statistical analyses were conducted using Stata, version 14 (StataCorp LLC). Hypothesis tests were 2 sided, and *P* < .05 was considered statistically significant. Given that the preintervention and postintervention survey populations were likely distinct, we assessed the differential effectiveness of the intervention by length of faculty appointment to better understand changing perceptions over time. We hypothesized that faculty appointed for 5 years or more would be more likely to have had a previous experience with lactation resources and be better poised to assess the effectiveness of the intervention compared with early faculty. To formally test whether the effectiveness of the intervention was modified by length of faculty appointment, we fit linear models with the outcome of satisfaction and with estimators that included a binary variable for intervention period, a binary variable for faculty appointment of 5 years or more, and an interaction between intervention period and length of faculty appointment. Initial data analysis was performed on February 22, 2022, with additional tests for interaction performed on May 18, 2023.

## Results

A total of 70 clinical faculty took childbearing leave in the 2-year preintervention period compared with 52 faculty in the postintervention period. The mean (SD) age of faculty taking childbearing leave was 34.4 (2.9) years in the preintervention period compared with 34.8 (2.7) years in the postintervention period. Faculty who took childbearing leave in the preintervention and postintervention periods represented all 19 clinical departments.

### Lactation wRVU Credit Program

In the first complete year of the program, 40 faculty took advantage of the lactation support intervention (Table 2). The program financially reimbursed $242 744.37 to these faculty, with an average payment of $6068 per faculty member. For the 15 faculty whose year following childbirth completely overlapped with the first year of the program and who remained at UCSF for the duration of that first year, the mean (SD) duration of lactation hold use was 8.9 (0.2) months. These faculty placed a mean (SD) of 84.9 (54.3) holds per faculty member (9.5 [5.1] per month), for an average reimbursement of $9125.78.

**Table 2.** Lactation Holds and wRVU Reimbursement for Ambulatory Clinicians Within the Study Year

Characteristic	All faculty taking leave within the study year (n = 40)	Faculty whose eligibility for wRVU credits fell completely within the study year (n = 15)
No. (%) of physicians in clinical setting		
Primary care	9 (22.5)	5 (33.3)
Medical subspecialty	12 (30.0)	2 (13.3)
Neurology	3 (7.5)	1 (6.7)
Surgery or surgical subspecialty	1 (2.5)	2 (13.3)
Ophthalmology	1 (2.5)	1 (6.7)
Obstetrics and gynecology	3 (7.5)	1 (6.7)
Pediatrics	2 (5.0)	NA
Pediatric subspecialty	6 (15.0)	2 (13.3)
Pediatric surgical or surgical subspecialty	1 (2.5)	NA
Procedural subspecialty (otolaryngology—head and neck surgery, orthopedics)	2 (5.0)	1 (6.7)
No. of lactation holds		
Total	2100	1273
Per faculty member, mean (SD)	52.5 (46.9)	84.9 (54.3)
Reimbursement, $		
Total	242 744.37	136 886.64
Per faculty member, average	6068.61	9125.78

Abbreviations: NA, not applicable; wRVU, work relative value unit.

### Room Allocation

In the first year of the improvement initiative, Campus Life Services opened 14 new lactation rooms with 19 total private stations, including 4 overnight call rooms repurposed into dual-use spaces that served as call rooms from 6 pm to 9 am and as pumping rooms from 10 am to 6 pm. Campus Life Services worked with hospitality services to ensure that all designated lactation rooms were cleaned twice daily. All rooms were evaluated for suitability of a desk, computer, and phone as well as for badge access and an internal lock for private spaces.

### Communication Regarding Resources and Policies

To address reported challenges in accessing information about pumping locations, how to reserve space, and finding information about available spaces in a limited time window, Campus Life Services expanded the number of rooms with online reservation functionality and implemented an email confirmation system to ensure the accuracy of existing reservations. In response to this initiative, numerous divisions and departments created custom lactation policies describing the resources available to their faculty and trainees both at the institutional and departmental levels (eAppendix 4 in Supplement 1).

### Faculty Satisfaction Survey Results

Of the 70 faculty surveyed, 58 (83%) responded to the preintervention survey; 54 of these individuals reported expressing milk and answered at least 1 survey question. A total of 48 faculty members responded to at least 1 survey question in the postintervention period, but the response rate was unknown due to the postimplementation survey methodology. Overall, there was no difference between the preintervention and postintervention periods in faculty support related to information, cleanliness, availability, proximity, and furnishings associated with lactation facilities. In the postintervention period, there was a significant increase in the reported support that faculty members received to devote time in their clinical schedule to pump (mean [SD] response, 2.5 [1.3] vs 3.6 [1.5]; *P* < .001) and around initiatives that addressed the impact of lactation time on productivity (mean [SD] response, 2.0 [1.0] vs 3.0 [1.5]; *P* < .001) (Table 3). Faculty were more likely to find the culture supportive of lactation in the postintervention period (mean [SD] response, 2.8 [1.4] vs 3.4 [1.3]; *P* = .047). No differences were observed among faculty who identified as working in the ambulatory, perioperative, or inpatient setting or in a mix of clinical locations.

**Table 3.** Faculty Satisfaction Related to Lactation Support Comparing Survey Answers Given During the Preintervention (Before or in July 2020) to Postintervention (After July 2020) Periods

Survey topic	Preintervention		Postintervention		*P* value
	Mean response (SD)	No. of faculty	Mean response (SD)	No. of faculty	
Level of support UCSF provided^a^					
Providing information about lactation accommodations	3.0 (1.4)	54	3.3 (1.4)	47	.33
Cleanliness of space for lactation	3.2 (1.5)	48	3.5 (1.3)	39	.29
Availability of space for lactation	3.0 (1.4)	52	3.4 (1.4)	44	.18
Proximity of space for lactation	2.9 (1.4)	51	3.1 (1.4)	43	.51
Lactation space furnishings and supplies (pumps, wipes, furniture, etc)	2.8 (1.4)	50	2.6 (1.4)	39	.45
Finding time in clinical schedule to devote to pumping	2.5 (1.3)	53	3.6 (1.5)	47	<.001
Initiatives to address impact of lactation time on productivity	2.0 (1.0)	50	3.0 (1.5)	44	<.001
Level of agreement^b^					
Supportive culture (messaging from supervisors and colleagues about time needed to pump)	2.8 (1.4)	54	3.4 (1.3)	46	.047
UCSF Health supports those who choose to pump	3.0 (1.1)	54	3.4 (1.0)	48	.08
Division or department supports those who choose to pump	3.4 (1.1)	54	3.7 (1.1)	48	.26

Abbreviation: UCSF, University of California San Francisco.

^a^ Five-point Likert scale from 1 (no support) to 5 (great support).

^b^ Five-point Likert scale from 1 (strongly disagree) to 5 (strongly agree).

A significant difference in lactation support was perceived when comparing faculty appointed for less than 5 years (Table 4) vs those appointed for 5 years or more (Table 5). In the subgroup of faculty appointed for 5 years or more, faculty support related to lactation information (mean [SD] response, 2.4 [1.5] vs 3.6 [1.2]; *P* = .02), cleanliness (mean [SD] response, 2.7 [1.4] vs 4.1 [1.0]; *P* = .007), availability (mean [SD] response, 2.7 [1.4] vs 4.4 [0.7]; *P* < .001), and proximity (mean [SD] response, 2.8 [1.5] vs 4.2 [0.9]; *P* = .008) associated with lactation facilities was significantly higher in the postintervention period. This subgroup also reported an increase in UCSF Health’s support of those who chose to pump (mean [SD] response, 2.6 [1.2] vs 3.5 [1.0]; *P* = .03). Using a linear model including an interaction between intervention period and length of faculty appointment, there was a statistically significant interaction between intervention time period and length of faculty appointment of practicing for 5 years or more for information provided (coefficient, 1.57; *P* = .007), cleanliness of lactation spaces (coefficient, 1.85; *P* = .004), availability of lactation spaces (coefficient, 1.92; *P* = .002), proximity to lactation spaces (coefficient, 1.72; *P* = .007), and furnishings within the lactation spaces (coefficient, 1.57; *P* = .02).

**Table 4.** Faculty Satisfaction Related to Lactation Support Comparing Survey Answers Given During the Preintervention (Before or in July 2020) to Postintervention (After July 2020) Periods, Restricted to Respondents Appointed for Less Than 5 Years

Survey topic	Preintervention		Postintervention		*P* value
	Mean response (SD)	No. of faculty	Mean response (SD)	No. of faculty	
Level of support UCSF provided^a^					
Providing information about lactation accommodations	3.6 (1.1)	27	3.1 (1.5)	34	.24
Cleanliness of space for lactation	3.7 (1.4)	23	3.3 (1.4)	29	.27
Availability of space for lactation	3.3 (1.2)	25	3.1 (1.5)	33	.48
Proximity of space for lactation	3.1 (1.3)	24	2.8 (1.4)	33	.42
Lactation space furnishings and supplies (pumps, wipes, furniture, etc)	3.2 (1.4)	24	2.4 (1.4)	30	.04
Finding time in clinical schedule to devote to pumping	2.7 (1.1)	27	3.5 (1.5)	34	.02
Initiatives to address impact of lactation time on productivity	2.3 (1.1)	25	2.9 (1.5)	32	.07
Level of agreement^b^					
Supportive culture (messaging from supervisors and colleagues about time needed to pump)	2.9 (1.5)	27	3.3 (1.4)	33	.22
UCSF Health supports those who choose to pump	3.4 (0.9)	27	3.4 (1.1)	35	.89
Division or department supports those who choose to pump	3.6 (1.0)	27	3.6 (1.1)	35	.79

Abbreviation: UCSF, University of California San Francisco.

^a^ Five-point Likert scale from 1 (no support) to 5 (great support).

^b^ Five-point Likert scale from 1 (strongly disagree) to 5 (strongly agree).

**Table 5.** Faculty Satisfaction With Lactation Support Comparing Survey Answers Given During the Preintervention (Before or in July 2020) to the Postintervention (After July 2020) Periods, Restricted to Respondents Appointed for 5 Years or More

Survey topic	Preintervention		Postintervention		*P* value
	Mean response (SD)	No. of faculty	Mean response (SD)	No. of faculty	
Level of support UCSF provided^a^					
Providing information about lactation accommodations	2.4 (1.5)	27	3.6 (1.2)	13	.02
Cleanliness of space for lactation	2.7 (1.4)	25	4.1 (1.0)	10	.007
Availability of space for lactation	2.7 (1.4)	27	4.4 (0.7)	11	<.001
Proximity of space for lactation	2.8 (1.5)	26	4.2 (0.9)	10	.008
Lactation space furnishings and supplies (pumps, wipes, furniture, etc)	2.5 (1.3)	27	3.2 (1.5)	9	.15
Finding time in clinical schedule to devote to pumping	2.3 (1.5)	26	3.8 (1.5)	13	.005
Initiatives to address impact of lactation time on productivity	1.7 (0.9)	25	3.1 (1.6)	13	.002
Level of agreement^b^					
Supportive culture (messaging from supervisors and colleagues about time needed to pump)	2.8 (1.4)	27	3.5 (1.3)	13	.10
UCSF Health supports those who choose to pump	2.6 (1.2)	27	3.5 (1.0)	13	.03
Division or department supports those who choose to pump	3.3 (1.2)	27	3.8 (1.0)	13	.19

Abbreviation: UCSF, University of California San Francisco.

^a^ Five-point Likert scale from 1 (no support) to 5 (great support).

^b^ Five-point Likert scale from 1 (strongly disagree) to 5 (strongly agree).

## Discussion

An initiative to improve lactation support for physicians returning from childbearing leave resulted in the creation of more numerous and functional lactation spaces, improvements in communication around lactation resources, and a program to reimburse faculty for time spent expressing breastmilk in the ambulatory setting. Forty faculty took advantage of the program and were reimbursed $242 744.37 for their time. Faculty who used the program for the entire year of the study placed lactation holds for a mean (SD) of 8.9 (0.2) months, with an average reimbursement of $9125.78. When comparing the preintervention to the postintervention period, faculty reported improvements in finding time in their clinical schedule to devote to pumping, initiatives to address the impact of lactation time on productivity, and a culture supportive of lactation. For those faculty at UCSF appointed for 5 years or more, improvements were also noted in providing information about lactation accommodations, cleanliness of space for lactation, availability of space for lactation, and the proximity of those spaces.

Nationwide surveys describe similar challenges to those faced by our faculty: namely, the lack of proximal, clean, and accessible locations for pumping breast milk; full patient loads, which make protecting time for pumping difficult; and the challenge of meeting productivity expectations.^5,14^ Previous studies have also acknowledged a culture not supportive of lactation, with one study finding that nearly one-third of pumping physicians experienced inappropriate comments or were made to feel uncomfortable for needing to pump.^14^ We observed that faculty appointed for 5 years or more perceived greater effectiveness of the intervention than those appointed for less than 5 years. Given the inability to account for repeated responses, it is possible that faculty appointed for less than 5 years who took the postimplementation survey were reporting on their initial lactation experience. Conversely, those appointed for longer than 5 years were more likely to have already experienced lactation at work with a previous birth prior to the intervention. Faculty appointed for 5 years or more were thus perhaps more reliably able to assess the effectiveness of the intervention compared with faculty who were only surveyed after their first lactation experience.

It is up to academic institutions to take up the baton and address lactation support as a real challenge to workplace equity and the advancement of women. The findings of this study suggest that institutions looking to support their female faculty need to invest in comprehensive and multifaceted lactation support programs that focus on proximal and functional private lactation spaces, flexible clinical schedules, and adjusted productivity expectations. Lactation support programs can benefit institutions through faculty retention and loyalty. Providing lactation support can help address physician mothers leaving the institution for a workplace that can accommodate lactation.

The challenges faced by lactating physicians are multifaceted and require a multipronged solution with an institutional commitment to build partnerships with physician leadership, finance, facilities, and hospitality. As noted in previous studies, changing culture requires discussing expectations around lactation accommodations with chairs, medical directors, and managers and consistently communicating lactation support policies to both leaders and those returning from childbearing leave.^15^ At our institution, we observed that increased dialogue about what is needed to support lactating physicians has helped to change the narrative around lactation and, in so doing, the institutional culture. These conversations with departmental groups and medical directors led to some benefits outside of our program. Departments have identified spaces for faculty to use outside of the official university lactation rooms and partnered with our lactation specialist in ensuring that rooms meet standard requirements. Several Department of Medicine inpatient services have developed a policy to reduce team caps for those with lactating physicians. The Department of Anesthesia provides lactating faculty break relief for pumping, outside of already scheduled breaks. The Department of Dermatology financially supports wearable pumps for their faculty and trainees. Numerous divisions and departments have created custom lactation policies describing the resources available to their faculty and trainees both at the institutional and departmental levels. Based on these successes, our campus lactation specialist now holds a quarterly seminar, “Managers for Workplace Lactation Support Toolkit,” to ensure that all leaders have the tools they need to support lactating faculty, trainees, and staff.

### Limitations

Our study has several limitations. First, as a single-site study, many of the features of our program (eg, the availability of a lactation specialist or an institutional financial commitment) may not be present. Due to our postimplementation survey methodology and the desire to reach inpatient and perioperative faculty who may not have used the ambulatory lactation credits, we were unable to calculate a response rate. Given the anonymous nature of the survey, we were unable to use paired *t* tests to examine perception improvement among physicians who might have had children before and after the implementation date. Finally, we did not capture the impressions of trainees, advanced practice practitioners, or staff who may have also benefited from improved lactation spaces, furnishings, cleanliness, and culture.

## Conclusions

In this cohort study of physicians returning from childbearing leave, a multifaceted intervention aimed at addressing common challenges in lactation support in an academic medical center was associated with modest improvements in faculty perceptions of institutional support for finding time to pump breastmilk, addressing the impact of lactation time on productivity, and providing a culture supportive of lactation. These interventions may serve as a model for other academic institutions seeking to improve physicians’ experience with lactation.

## Notes

Supplement 1. **eAppendix 1.** Lactation Work Relative Value Unit (wRVU) Credit Cost Estimate **eAppendix 2.** Lactation Work Relative Value Unit (wRVU) Credit Program Publicity **eAppendix 3.** Preintervention and Postintervention Survey **eAppendix 4.** Departmental Lactation Policy Template

Supplement 2.
Data Sharing Statement
