# Supplementary material for: Physician Satisfaction With Lactation Resources Following an Intervention to Improve Lactation Accommodations
Source: JAMA Netw Open. 2023 Aug 8;6(8):e2327757. doi: 10.1001/jamanetworkopen.2023.27757 (PMC10410473; doi:10.1001/jamanetworkopen.2023.27757)
Supplement: Supplement 1. — eAppendix 1. Lactation Work Relative Value Unit (wRVU) Credit Cost Estimate eAppendix 2. Lactation Work Relative Value Unit (wRVU) Credit Program Publicity eAppendix 3. Preintervention and Postintervention Survey eAppendix 4. Departmental Lactation Policy Template [file jamanetwopen-e2327757-s001.pdf]

Supplementary Online Content

Mourad M, Prasad P, Wick C, Sliwka D. Physician satisfaction with lactation resources following an intervention to improve lactation accommodations. *JAMA Netw Open*. 2023;6(8):e2327757. doi:10.1001/jamanetworkopen.2023.27757

eAppendix 1. Lactation Work Relative Value Unit (wRVU) Credit Cost Estimate

eAppendix 2. Lactation Work Relative Value Unit (wRVU) Credit Program Publicity

eAppendix 3. Preintervention and Postintervention Survey

eAppendix 4. Departmental Lactation Policy Template

This supplementary material has been provided by the authors to give readers additional information about their work.

© 2023 Mourad M et al. *JAMA Network Open*.

eAppendix 1. Lactation Work Relative Value Unit (wRVU) Credit Cost Estimate

Cost Estimate

To estimate the costs of a lactation wRVU support program, we partnered with Human Resources to obtain consent from physicians who took childbearing leave from July 2018 – June 2020 to share their department, division, and leave dates. Of 87 physicians contacted, 82 provided their consent, We included the 36 physicians with ambulatory clinical roles whose leave dates spanned July 2019 – June 2020. To estimate the costs of reimbursing physicians for time spent lactating we assumed 1) All physicians would use 30 minutes lactation time per clinic session for 9 months after their return from leave and 2) Their ambulatory clinical time after their leave would be the same as prior to leave. Together with the faculty medical group financial team, we used data on the number of clinic sessions and earnings per clinic session to estimate the costs of supporting 1/8th of their ambulatory time for 9 months. We estimated an annual cost of $325,000 to support physician time spent lactating.

© 2023 Mourad M et al. *JAMA Network Open*.

eAppendix 2. Lactation Work Relative Value Unit (wRVU) Credit Program Publicity

Lactation wRVU Credit Program Publicity

At the initiation of the program, we presented the details of the program to all medical directors and clinic administrators. We created a detailed FAQ and tip sheet on the program eligibility and posted both on our institutional Lactation Accommodation Program website. All ambulatory physicians with a childbearing leave in the preceding 12 months were notified about the start of the program with instructions on how to correctly place lactation holds into their clinical schedule. We encouraged them to work with their ambulatory clinic schedulers to build the holds into their 4-hour clinic sessions. We worked with clinic directors, department administrators and human resources to ensure advertisement of the program to their faculty moving forward.

© 2023 Mourad M et al. *JAMA Network Open*.

eAppendix 3. Preintervention and Postintervention Survey

Lactation Experience Survey

Q1 Return from parental leave is a challenging time for faculty and associated with increase work responsibilities at home, challenges in navigating child care, and sleep deprivation. For many faculty the desire to continue breast feeding and integrating that into a busy work schedule presents an additional struggle.

Despite excellent lactation initiation rates, lactating physicians are at risk of premature lactation cessation, citing lack of sufficient time and adequate place for milk expression at work. Although not all lactating parents identify as women, this problem disproportionately impacts women at work and creates inequity that contributes to the gender gap in work experience.   You are receiving this survey because you _may have_ returned from childbearing and _may have_ used UCSF Lactation support services. We are conducting a survey to understand your experience, with the goal of reducing barriers to pumping at work.

With questions regarding/ feedback on this survey, please contact Michelle.Mourad@ucsf.edu

End of Block: Default Question Block

Start of Block: Block 1

Q2 Did you express milk while at work following return from child-bearing leave?

○ Yes (1)

○ No (2)

Skip To: End of Survey If Did you express milk while at work following return from child-bearing leave? = No

End of Block: Block 1

Start of Block: Block 2

© 2023 Mourad M et al. *JAMA Network Open*.

Q3 If you expressed milk while at work following child-bearing leave at UCSF, at the time, how did UCSF support your experience in the following domains (if you have had two children while at UCSF, please answer thinking about your most recent experience):

	Provided no support (1)	Not supportive (2)	Neutral (3)	A small amount of support (4)	Provided great support (5)	N/A (6)
Providing information about lactation accommodations (e.g. UCSF policies, pumping locations and available equipment) (1)	○	○	○	○	○	○
Cleanliness of space for lactation (4)	○	○	○	○	○	○
Availability of space for lactation (5)	○	○	○	○	○	○
Proximity of space for lactation (6)	○	○	○	○	○	○
Lactation space furnishings and supplies (pumps, wipes, furniture, phones, etc.) (7)	○	○	○	○	○	○
Finding time in your clinical schedule to devote to pumping (8)	○	○	○	○	○	○
Initiatives to address the impact of lactation time on your clinical productivity (9)	○	○	○	○	○	○
Supportive Culture (messaging from supervisors and colleagues about time needed to pump) (10)	○	○	○	○	○	○

End of Block: Block 2

Start of Block: Block 3

© 2023 Mourad M et al. *JAMA Network Open*.

Q9 Provide a rating for the statement below.

	Strongly disagree (1)	Disagree (2)	Neither agree nor disagree (3)	Agree (4)	Strongly agree (5)
UCSF Health supports those returning from childbearing leave who choose to pump. (1)	○	○	○	○	○

End of Block: Block 3

Start of Block: Block 4

Q11 Provide a rating for the statement below.

	Strongly disagree (1)	Disagree (2)	Neither agree nor disagree (3)	Agree (4)	Strongly agree (5)
My Division/Department supports those returning from childbearing leave who choose to pump. (1)	○	○	○	○	○

End of Block: Block 4

Start of Block: Block 5

© 2023 Mourad M et al. *JAMA Network Open*.

Q6 How likely are you to recommend UCSF as a place to work as a lactating parent: 0-10 scale (11 points)

- ○ 0 (0)
- ○ 1 (1)
- ○ 2 (2)
- ○ 3 (3)
- ○ 4 (4)
- ○ 5 (5)
- ○ 6 (6)
- ○ 7 (7)
- ○ 8 (8)
- ○ 9 (9)
- ○ 10 (10)

Q14 What is working well with Lactation support at UCSF?

____

____

____

____

____

End of Block: Block 5

Start of Block: Block 6

Q4 Please provide your top priorities in improving the Lactation experience at UCSF in the future.

____

____

____

____

____

End of Block: Block 6

Start of Block: Block 7

Q8 Department:

▼ Anesthesia (4) ... Urology (42)

© 2023 Mourad M et al. *JAMA Network Open*.

Q9 Primary Setting of Clinical Work:

- Ambulatory (1)
- Inpatient (2)
- Periop (4)
- Mixture of more than one area (5) ____

Q15 Primary site for clinical care

- UCSF Health West Bay based sites (Parnassus, MZ, Mission Bay, San Mateo etc.) (1)
- UCSF East Bay (e.g. BCH Oakland, Berkley Outpatient Center) (4)
- ZSFG (2)
- SF VA (3)

Display This Question:
If Primary site for clinical care = UCSF Health West Bay based sites (Parnassus, MZ, Mission Bay, San Mateo etc.)

Q10 Primary Location of Clinical Work:

- China Basin (1)
- Laurel Village (4)
- Lakeshore (5)
- Mission Bay (6)
- Mount Zion (7)
- San Mateo (8)
- Parnassus (9)
- Other (3) ____

© 2023 Mourad M et al. *JAMA Network Open*.

Q11 Years on Faculty:

- 0-2 (1)
- 3-5 (4)
- 5-10 (5)
- 10+ (6)

End of Block: Block 7

© 2023 Mourad M et al. *JAMA Network Open*.

eAppendix 4. Departmental Lactation Policy Template

[Department] Lactation Accommodation Program Support and Guidelines

Congratulations on the upcoming addition to your family, or if you are returning from leave, on your new little one! These guidelines are to support you if plan to breastfeed your baby and pump milk when you return to work. If you have any questions, please reach out to name@ucsf.edu. In this guide you will find the following

Institutional Resources:

1. List of available facilities and access to hospital grade pumps: http://tiny.ucsf.edu/lactationrooms
2. How to register for the UCSF Lactation Accommodation Program and obtain a free cooler bag: http://tiny.ucsf.edu/lactationregister
3. Information on obtaining badge access to lactation rooms: lactationprogram@ucsf.edu
4. Who to contact for room cleaning/functionality: lactationprogram@ucsf.edu
5. Departmental Contact: name@ucsf.edu.
6. Instructions for seeking formal accommodations. If larger schedule modifications are required, contact Disability-Management Services.

Common Departmental Lactation Spaces:

Below are the primary spaces for Departmental faculty and staff while either on service at any of our hospitals, or doing administrative work. Please also check the lactation program's website, here, for the most recent updates.

Building:

- Dual Lactation/Call Rooms:
  - Room #, Code or Badge Access, Hours
  - Pump present in Room (Y/N)
  - Desk/Computer/phone present in room (Y/N)
  - Sink present in room (Y/N)
  - Nearest fridge access:

Department Pumping Supplies/Equipment:

- Refrigerator Locations:
- All lactating parents are entitled to a free cooler from the Lactation Accommodation Program, which can be secured by emailing lactationprogram@ucsf.edu.
- As noted above, many lactation rooms at UCSF Health are equipped with the hospital-grade Ameda Elite pump. You can purchase compatible pump parts on your own, or through the Mission Bay Friend to Friend gifts shop. If you forget your pump parts one day, you can contact either the Mission Bay gift shop (415-353-7776) or the Parnassus Gift Shop (415-353-1845).

© 2023 Mourad M et al. *JAMA Network Open*.

Cinical Accommodation:

The [Department] will provide accommodations during clinical work to faculty to allow for time to pump, as noted below.

Inpatient Accommodations:

UCSF Ambulatory Lactation Credit:

UCSF Health provides lactation hold blocks on provider schedule templates for returning moms. A provider can have one lactation hold per 4-hour session. With the placement of each hold, faculty will receive credit equal to 99214 (currently 1.92 wRVU) that will be part of Funds Flow payments to the Department/Division on a quarterly basis.

To receive the wRVU credit, you need to set your scheduling template up with the lactation holds. Please contact your clinic scheduling team to add the hold to your schedule, or see Appendix 1 with detailed instructions

Administrative Accommodations:

Lactating faculty, staff, and fellows should block off time for pumping into their administrative schedules, which is separate from lunch breaks. A reasonable amount of time for a lactation break is generally considered 30 minutes every 2-4 hours, which includes the time associated with travel to and from the lactation space, milk expression, clean-up, and storage. We recommend that these times be blocked on individual's Outlook calendars to ensure meetings are not scheduled during lactation times.

© 2023 Mourad M et al. *JAMA Network Open*.
