# Supplementary material for: Physician Satisfaction With Lactation Resources Following an Intervention to Improve Lactation Accommodations
Source: JAMA Netw Open. 2023 Aug 8;6(8):e2327757. doi: 10.1001/jamanetworkopen.2023.27757 (PMC10410473; doi:10.1001/jamanetworkopen.2023.27757)
Supplement: Supplement 2. — Data Sharing Statement [file jamanetwopen-e2327757-s002.pdf]

Data Sharing Statement

Mourad. Physician Satisfaction With Lactation Resources Following an Intervention to Improve Lactation Accommodations. *JAMA Netw Open*. Published August 08, 2023. doi:10.1001/jamanetworkopen.2023.27757

Data
Data available: No

Additional Information
Explanation for why data not available: Contains sensitive HR data, which would be hard to completely de-identify
